# Metformin-Induced Chronic Diarrhea Misdiagnosed as Irritable Bowel Syndrome for Years

**DOI:** 10.7759/cureus.56828

**Published:** 2024-03-24

**Authors:** Monika Chaudhary, Naresh K Midha, Pankaj Sukhadiya, Deepak Kumar, Mahendra Kumar Garg

**Affiliations:** 1 General Medicine, All India Institute of Medical Sciences, Jodhpur, IND

**Keywords:** metformin-associated gastrointestinal intolerance, irritable bowel syndrome, oral hypoglycemic drugs (ohas), chronic diarrhoea, irritable bowel disorder, diabetes mellitus

## Abstract

Metformin is the most prescribed and recommended drug for type 2 diabetes mellitus because of its better tolerability, pleiotropic benefits, and cost-effectiveness. Metformin inhibits hepatic glucose production and increases muscle glucose uptake. Metformin is also associated with gastrointestinal side effects like abdominal bloating, flatulence, diarrhea, nausea, and vomiting. Metformin-related gastrointestinal side effects are mainly due to alteration in gut microbiota, raised intestinal glucose, and increased ileal bile salt reabsorption. We report a case of a 62-year-old diabetic patient who presented with chronic diarrhea with a weight loss of 6 kg from the last six years after initiation of metformin. He underwent multiple investigations and was finally misdiagnosed with irritable bowel syndrome for years. After discontinuation of metformin, there was a significant improvement in gastrointestinal symptoms. Our case highlights the importance of metformin-induced chronic diarrhea if no other causes for the diarrhea are obvious in patients with type 2 diabetes taking metformin. Consideration of this potential side effect of metformin must be valuable to avoid unwarranted investigations, additional drug therapy, and annoyance of the patients.

## Introduction

Metformin is a biguanide-type oral hypoglycemic drug that is universally prescribed for type 2 diabetes mellitus. Metformin is recommended as a first-line drug for type 2 diabetes because of its safety, tolerability, reasonable cost, and possible cardiovascular benefits. Metformin decreases blood glucose by inhibiting hepatic gluconeogenesis and increasing skeletal muscle uptake of glucose. Metformin is also well known for its gastrointestinal (GI) side effects, such as abdominal bloating, flatulence, diarrhea, nausea, and vomiting [1].

Metformin-associated GI side effects are common in approximately 20% of patients. These adverse effects significantly impact treatment adherence, prompting some individuals to discontinue medication and experience a decline in their health-related quality of life [2]. These GI side effects often diminish over time and can be minimized by careful dose adjustment and taking metformin with meals. Approximately 5% of patients discontinue metformin due to these side effects [3].

Metformin-induced GI intolerance is explained by exerting effects on gut microbiota, increasing intestinal glucose turnover, alteration in bile salt reabsorption, intestinal lactate production, and increasing glucagon-like peptide 1 (GLP-1) concentration [4].

We report a case of chronic diarrhea with weight loss in a 62-year-old male patient with type 2 diabetes who was taking sustained-release metformin 1000 mg/day for six years. He endured multiple investigations and was misdiagnosed as irritable bowel syndrome for years. After discontinuation of metformin, there was a significant improvement in GI symptoms. Our case emphasizes the consideration of metformin-induced chronic diarrhea if no other causes for the diarrhea are obvious in the patients with type 2 diabetes taking metformin. Awareness of this potential side effect of metformin may help reduce the suffering of the patients and the cost of diagnostic procedures.

## Case presentation

A 62-year-old male was diagnosed with type 2 diabetes mellitus seven years back. He presented with complaints of chronic diarrhea with unintentional weight loss of 6 to 8 kg for six years. He complained of an increased frequency of painless, partially formed stools with abdominal bloating and cramping. The patient was having nocturnal symptoms in the form of abdominal bloating associated with the increased urge to defecate. These abdominal symptoms usually occur only after meals. He had a history of unintentional weight loss of 6 to 8 kg without any constitutional symptoms of low-grade fever or decreased appetite. He reported well-controlled diabetes with sustained-release metformin 500 mg twice a day. He was taking atorvastatin 20 mg once a day for hypercholesterolemia and telmisartan 40 mg once a day for hypertension. He refuted any previous history of celiac disease or lactose intolerance. He denied the use of any Ayurvedic or herbal medication for diabetes. He visited a primary care physician, gastroenterologist, and psychiatrist for the above complaints. An extensive workup was already done for diabetic gastroparesis, inflammatory bowel disease, and infective pathology. Ultimately, he was diagnosed with irritable bowel syndrome diarrhea type, and weight loss was considered due to metformin and strict diet control. The patient was treated with probiotics, amitriptyline, and antibiotics like rifaximin and metronidazole. Despite this treatment, the patient did not get any improvement and he finally visited our outpatient department.

On the first OPD visit, he was found normotensive, with a BMI of 22.5 kg/m2. General physical and systemic examinations were unremarkable. We evaluated his previous investigations, which showed a mild increase in C-reactive protein (CRP, 3.0 mg/dL) and a normal colonoscopy and CT scan of his abdomen. We planned a battery of screening laboratory investigations, which showed normal liver and kidney functions with hemoglobin of 14.4 gm% and glycosylated hemoglobin of 7.2%. Inflammatory markers (CRP and erythrocyte sedimentation rate), thyroid functions, and lipid profile were normal. Stool microscopy for ova and parasites was found negative. The abdominal ultrasound was also unremarkable. On this first consultation, after careful evaluation of all previous and current laboratory reports, we framed two primary differentials, i.e., drug-induced chronic diarrhea and any potential infective colitis. Although we did not find any clear signs of infection, the patient's weight loss was noticeable. We stopped atorvastatin and telmisartan because the lipid profile was normal, and the patient was normotensive. The patient was advised to have dietary controls and regular blood pressure monitoring. We started the probiotic *Saccharomyces boulardii* (250 mg) for 10 days. The patient was re-evaluated after 10 days. There was mild improvement in his symptoms on the second OPD visit. In the dearth of any evidence of infective pathology, we came to the diagnosis of metformin-induced chronic diarrhea and we stopped metformin for one month. Metformin was substituted with glimepiride 0.5 mg twice a day. The patient was scheduled for a follow-up colonoscopy and biopsy in one month if symptoms persisted. Fortunately, there was a notable improvement in diarrhea and bloating within this timeframe, obviating the need for further intervention.

## Discussion

Chronic diarrhea is a common problem in diabetic patients. The prevalence of diarrhea in diabetic patients is estimated at approximately 3.7-22%. This is because of various reasons like concomitant endocrine disorders, autonomic dysfunction, celiac disease, chronic pancreatitis, or small intestinal bacterial overgrowth (SIBO) [5].

Type 2 diabetic patients are taking multiple medications commonly, and drug-induced diarrhea is a considerable cause in these patients. Combination of metformin with dipeptidyl peptidase IV (DPP IV) inhibitors, alpha-glucosidase inhibitors (e.g., acarbose), and GLP-1 receptor agonists are associated with higher GI side effects than metformin monotherapy [6].

Metformin-induced diarrhea is mainly due to changes in the gut microbiome, increased intestinal glucose and bile acid turnover, and increased GLP-1 concentration. Decreased ileal bile salt reabsorption enhances the secretory effect of bile salts in the colon and increases intestinal motility with malabsorption [7]. Metformin-induced weight loss has been postulated as the result of alteration in leptin-associated adipocyte metabolism as well as a reduction of carbohydrate absorption from the gut [8].

Certain comorbidities and medications may also affect the GI tolerability of metformin. Chronic gastritis, *Helicobacter pylori* infection, recent GI surgeries, and drugs like proton-pump inhibitors are associated with a higher risk of metformin-induced GI side effects [9].

Recent studies have shown the role of a highly polymorphic organic cation transporter 1 (OCT1) in metformin absorption and metabolism. In OCT1-deficient individuals, reduced metformin absorption and aggravated GI intolerance are mentioned [10].

In our case report, there were no evident causing factors such as autonomic gastropathy or any significant comorbidity. Alteration in gut microbiota due to elderly age and drugs may have an important role in metformin-induced GI intolerance.

## Conclusions

Chronic diarrhea associated with metformin can occur in patients with type 2 diabetes after taking metformin for several years. Awareness of this complication and routine questioning of patients taking metformin would be helpful to prevent unnecessary investigations, additional drug therapy, and patient discomfort and inconvenience.
